# One-Minute Sit-to-Stand Test Versus Six-Minute-Walk Test in Post-COVID-19 Patients: A Cross-Sectional Observational Study

**DOI:** 10.3390/jcm15072479

**Published:** 2026-03-24

**Authors:** Marta Duarte-Silva, Pedro Fiúza, Neuza Reis, Miguel Toscano-Rico

**Affiliations:** 1 Clínica de Atendimento Post-COVID, ULS São José, 1169-024 Lisboa, Portugal; neuza.reis@ulssjose.min-saude.pt (N.R.); miguel.rico@ulssjose.min-saude.pt (M.T.-R.); 2 Department of Respiratory Medicine, Santa Marta Hospital, ULS São José, 1169-024 Lisboa, Portugal; 3 Department of Internal Medicine, Santa Marta Hospital, ULS São José, 1169-024 Lisboa, Portugal

**Keywords:** COVID-19, 1MSTST, 6MWT

## Abstract

**Background:** Simplified field tests have gained increasing interest for the assessment of functional capacity in patients with post-COVID-19 condition; however, direct comparisons of functional performance and physiological responses between the 1-min sit-to-stand test (1MSTST) and the 6-min walk test (6MWT) remain limited. This study aimed to examine the associations between the two tests by evaluating functional performance, cardiopulmonary responses, oxygen desaturation, perceived exertion, and peripheral muscle strength. Furthermore, we explored whether the 1MSTST can be used as a complementary assessment, particularly within telerehabilitation pathways and in contexts where resource-intensive testing is not feasible. **Methods:** We conducted a cross-sectional observational study of adults recovering from moderate to severe COVID-19 between May and July 2021. Participants performed both the 1MSTST and 6MWT on the same day. Functional performance, peak heart rate, nadir peripheral oxygen saturation (SpO_2_), perceived exertion, and handgrip dynamometry were recorded. Associations between test performances were assessed using correlation and partial correlation analyses, including adjustment for peripheral muscle strength. **Results:** Fifty-four patients were included. A moderate correlation was observed between 1MSTST repetitions and 6MWT distance (Spearman’s ρ = 0.47, *p* < 0.001), which was attenuated after adjustment for muscle strength and demographic variables. Peak heart rate and nadir SpO_2_ responses were strongly correlated between tests (r = 0.75 and ρ = 0.83, respectively; both *p* < 0.001), with no significant differences in magnitude. Exercise-induced oxygen desaturation (≥4% SpO_2_ drop) occurred at similar frequencies during both tests. Perceived exertion increased similarly following the 1MSTST and the 6MWT. **Conclusions**: In post-COVID-19 patients, the 1 min sit-to-stand test shows moderate concordance with the 6 min walk test for functional performance and strong agreement in cardiopulmonary responses. These findings suggest that the two tests assess overlapping but distinct aspects of functional capacity. This supports the use of the 1MSTST as a pragmatic complementary assessment when standard walking tests are not feasible, particularly within telerehabilitation pathways, primary care, and resource-limited settings.

## 1. Introduction

The coronavirus disease 2019 (COVID-19) pandemic has resulted in a growing population of individuals experiencing persistent symptoms and functional limitations long after recovery from the acute infection. A substantial proportion of survivors develop post-acute sequelae of SARS-CoV-2 infection (PASC), commonly referred to as long COVID, characterized by ongoing fatigue, exertional dyspnoea, reduced exercise tolerance, and impaired quality of life [1].

Growing longitudinal evidence indicates that traditional resting assessments alone may be insufficient to capture post-COVID-19 impairment. Several prospective studies have shown that pulmonary function parameters such as forced vital capacity and diffusion capacity may remain stable over time despite persistent symptoms. In a large prospective cohort study with structured follow-up at 3 and 6 months after acute COVID-19, Freund et al. demonstrated that routine pulmonary function measures did not consistently change over time and were poorly correlated with radiological improvement, highlighting the limited sensitivity of standard evaluations and the need for additional functional testing in selected patient populations [2]. Consistent with these findings, systematic reviews and longitudinal cohorts have reported persistent reductions in exercise capacity and physical performance months after COVID-19, even when resting lung function appears normal. Objective functional tests are therefore increasingly recognized as important components of post-COVID-19 follow-up and rehabilitation strategies [3,4].

The 6-min walk test (6MWT) is widely regarded as a standard field test for the evaluation of functional exercise capacity and exertional oxygen desaturation in patients with cardiopulmonary disease. It is a simple, inexpensive, and well-standardized test with established reference equations and broad use in clinical practice [5]. The primary outcome of the test is the total distance walked over 6 min (6MWD), which provides clinically relevant information on submaximal exercise capacity and gas-exchange abnormalities. However, the execution of the 6MWT requires a corridor of 20–30 m and strict methodological standardization, which may limit its feasibility in outpatient, primary care, or resource-limited settings.

In the context of COVID-19, the 6MWT is recommended as a screening tool in the British Thoracic Society guidance for respiratory follow-up, particularly in patients with persistent respiratory symptoms and/or incomplete radiographic resolution [6]. Several studies have demonstrated reduced 6MWT performance in post-COVID-19 patients months after hospital discharge, particularly in those with greater comorbidity burden, prior intensive care admission, or residual pulmonary abnormalities on chest imaging [5,7]. Exercise-induced desaturation during the 6MWT has also been associated with poorer pulmonary function and greater structural lung involvement, reinforcing its role as a tool to identify patients with more pronounced functional impairment [8].

The sit-to-stand test (STST) represents a complementary field test designed to assess physical performance in individuals for whom walking-based assessments may be impractical. The STST requires participants to repeatedly stand up from and sit down on a chair for a defined period and exists in several formats, including the five-repetition, 30 s, and 1-min versions. Among these, the 1-min sit-to-stand test (1-min STST) has demonstrated the strongest association with walking-based functional tests, including the 6MWT, in chronic respiratory diseases such as chronic obstructive pulmonary disease, interstitial lung diseases, and pulmonary hypertension [9,10,11]. Although the 1 min STST places greater mechanical demand on the lower limbs and may stress muscular endurance to a greater extent, it provides valuable information on overall physical capacity and exercise-induced desaturation while requiring minimal space, equipment, and time [5].

In post-COVID-19 populations, the 1 min STST has been shown to be feasible, safe, have excellent test–retest reliability and sensitivity to functional impairment, with a substantial proportion of patients demonstrating reduced performance relative to reference values and clinically relevant oxygen desaturation during the test [5,12]. Importantly, the 1 min STST exhibits minimal learning effect and can be performed as a single trial, making it particularly suitable for outpatient assessment, rehabilitation planning, and telehealth-based monitoring [13].

Despite growing interest in simplified field tests for the assessment of functional capacity, direct comparisons of functional performance and physiological responses between the 1 min sit-to-stand test (1MSTST) and the 6 min walk test (6MWT) in adults recovering from acute COVID-19 remain limited. The present study aimed to examine the relationship between the 1MSTST and the 6MWT in post-COVID-19 patients by evaluating associations with test performance, cardiopulmonary responses, oxygen desaturation, perceived exertion, and peripheral muscle strength. Specifically, we sought to determine whether the 1MSTST elicits physiological and perceptual responses comparable to those of the 6MWT, thereby supporting its use as a complementary, pragmatic assessment particularly within telerehabilitation pathways and when logistical constraints limit the feasibility of standard testing, notably in primary care and resource-limited environments. 

## 2. Materials and Methods

We conducted a cross-sectional observational study in adult patients with post-COVID-19 condition who had experienced moderate to severe acute SARS-CoV-2 infection and were referred for follow-up to the Post-COVID Outpatient Clinic (CAP-COVID) of Unidade Local de Saúde (ULS) de São José, Lisbon, Portugal. CAP-COVID is a multidisciplinary clinic dedicated to the integrated management of patients with acute sequelae and long COVID, involving specialists from internal medicine, pulmonology, physical and rehabilitation medicine, neurology, psychiatry, and nutrition. At the time of the study, follow-up assessments were coordinated through the Department of Internal Medicine. Participants were recruited between May and July 2021. Upon admission to the clinic, written informed consent was obtained, and patients underwent standardized clinical evaluation.

The severity of the acute SARS-CoV-2 infection was classified according to the Portuguese Directorate-General of Health (DGS) guideline (Norma n° 004/2020) [14]. To assess recovery after SARS-CoV-2 infection (including changes in lifestyle, sports, and social activities), the post-COVID-19 Functional Status (PCFS) scale was used. This is a validated ordinal scale developed to quantify functional limitations following COVID-19 infection, ranging from 0 (no functional limitations) to 4 (severe functional limitations) [15,16]. Functional exercise capacity was evaluated using both the 1 min sit-to-stand test (1MSTST) and the 6 min walk test (6MWT). Both tests were performed on the same day, separated by a 1-h rest interval, and tested by the same trained examiner. Participants received standardized instructions prior to each test, without verbal encouragement during performance.

The 1MSTST was conducted using a standardized chair (seat height 46 cm) without armrests. Participants were instructed to stand up and sit down as many times as possible within 1 min, and the total number of completed repetitions was recorded. The 6MWT was performed in accordance with American Thoracic Society guidelines, using a flat, straight 30 m indoor corridor [16]. The total distance walked over 6 min was recorded. For both tests, systolic and diastolic blood pressure were measured immediately before and after exercise. Heart rate and peripheral oxygen saturation (SpO_2_) were continuously monitored from 2 min before to 3 min after test completion. Perceived exertion was assessed using the Modified Borg scale immediately before and after each test. Handgrip strength was measured as an index of peripheral muscle strength using a Jamar^®^ hydraulic hand dynamometer, following standardized procedures.

A total of 54 patients completed both the 1MSTST and the 6MWT and were included in the final analysis. Participant inclusion was contingent on the availability of hospital staff to administer both tests on the same day. Exclusion criteria are detailed in Table 1. The study was conducted in accordance with the Declaration of Helsinki and was approved by the Ethics Committee of ULS São José, Lisbon, Portugal (approval no. 1209/2022). Patient recruitment and data collection were performed under the institutional framework established for COVID-19 research during the pandemic, regulated by an institutional circular on personal data protection and consent procedures (Circular Informativa No. 160/2020). This framework allowed the collection and processing of personal and clinical data for observational COVID-19–related research, provided that participants gave informed consent and that data protection procedures were respected.

### Statistical Analysis

Sample size determination was based on the detection of a 0.45 correlation coefficient between both tests with a power of 90% and an alpha level of 0.05. The number of participants required for the study was determined to be 47. Continuous variables were expressed as mean ± standard deviation (SD) when normally distributed and as median (interquartile range, IQR) when non-normally distributed. Categorical variables were summarized as frequencies and percentages. Normality was assessed using the Shapiro–Wilk test.

The association between functional performance in the 1 min sit-to-stand test (1MSTST; repetitions) and the 6 min walk test (6MWT; distance in meters) was evaluated using Spearman’s rank correlation coefficient. Peak heart rate (HR) responses during the two tests were compared using a paired-samples *t*-test, and their association was assessed using Pearson’s correlation coefficient. Nadir peripheral oxygen saturation (SpO_2_) values were compared using the Wilcoxon signed-rank test, and associations between SpO_2_ values across tests were evaluated using Spearman’s rank correlation. Exercise-related oxygen desaturation was defined as a ≥4% decrease in SpO_2_ from resting values. The proportion of participants meeting this criterion during each test was calculated, and paired comparisons were performed using McNemar’s test. Perceived dyspnoea (Modified Borg scale) changes from rest to post-test were analyzed separately for each test using Wilcoxon signed-rank tests. Modified Borg scale scores obtained after the 1MSTST and 6MWT were compared using a Wilcoxon signed-rank test, and their association was assessed using Spearman’s rank correlation coefficient. For non-parametric paired comparisons, effect size was calculated as *r* = *Z*/√N and interpreted as small (r ≈ 0.1), moderate (r ≈ 0.3), or large (r ≥ 0.5). Partial correlation analyses were conducted to explore the influence of peripheral muscle strength (handgrip dynamometry) on the association between functional test outcomes. Additional exploratory analyses controlling for age, sex, and post-COVID-19 functional status (PCFS) were performed to assess the robustness of observed associations.

All tests were two-tailed, and a *p*-value < 0.05 was considered statistically significant. Statistical analyses were performed using IBM SPSS Statistics (version 31.0.0.0 (117)).

## 3. Results

A total of 54 patients completed both the 1 min sit-to-stand test (1MSTST) and the 6 min walk test (6MWT) and were included in the analysis. The mean age of the cohort was 57.9 ± 12.5 years, and 29 participants (54%) were male. Most patients had experienced a moderate SARS-CoV-2 infection (n = 32, 59%). The most frequently observed post-COVID-19 functional status category was PCFS 2 (slight functional limitation), accounting for 42.6% of participants. The mean time from acute infection to evaluation was 3.0 ± 2.4 months. The mean number of repetitions achieved during the 1MSTST was 20.5 ± 6.1, and the mean distance walked during the 6MWT was 412 ± 90 m (Table 2).

**Figure 1:**
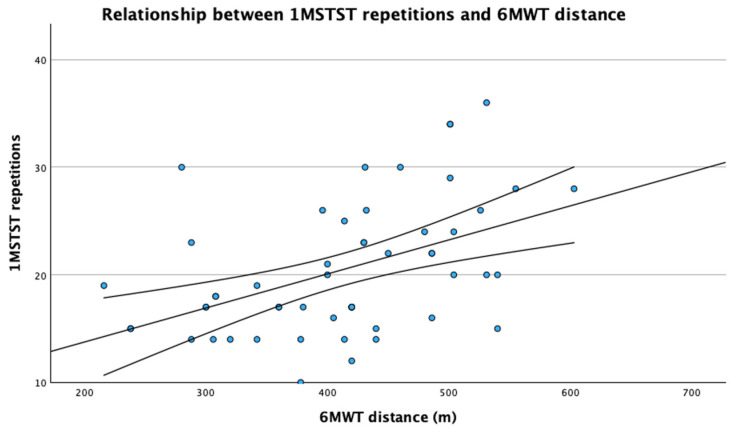
Relationship between 1MSTST repetitions and 6MWT distance. Each point represents an individual participant. The solid line indicates the fitted linear trend, and the outer lines represent the 95% confidence interval of the mean predicted value. A moderate association was observed between 1MSTST repetitions and 6MWT distance (Spearman’s ρ = 0.47, *p* < 0.001).

A moderate positive association was observed between the number of repetitions achieved during the 1MSTST and the distance walked during the 6MWT (Spearman’s ρ = 0.47, *p* < 0.001; Figure 1). Peripheral muscle strength assessed by hand grip dynamometry was positively correlated with performance in both the 1MSTST (ρ = 0.49, *p* < 0.001) and the 6MWT (ρ = 0.41, *p* = 0.003). When controlling for peripheral muscle strength, the association between 1MSTST repetitions and 6MWT distance was attenuated but remained statistically significant (partial r = 0.35, *p* = 0.012), although the magnitude of the association was smaller than that assumed for sample size estimation. Further adjustment for age, sex, and post-COVID-19 functional status resulted in additional attenuation of the association, which was no longer statistically significant (partial r = 0.26, *p* = 0.070) (Table 3).

**Figure 2:**
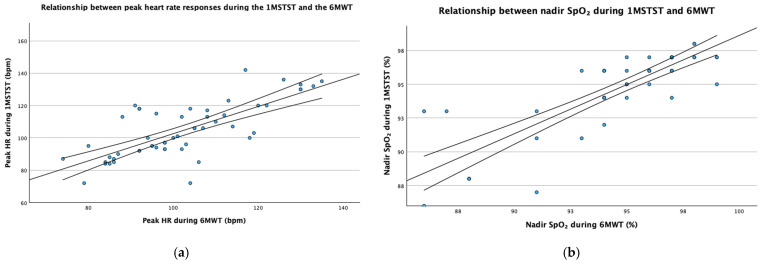
(**a**,**b**) Relationship between peak heart rate responses and SpO_2_ nadir values during the 1 min sit-to-stand test (1MSTST) and the 6 min walk test (6MWT). Each point represents an individual participant. The solid line represents the fitted linear trend; outer lines indicate the 95% confidence interval of the mean predicted value. Peak heart rate responses were strongly correlated between tests (Pearson’s r = 0.75, *p* < 0.001). Nadir SpO_2_ values were also strongly correlated between tests (Spearman’s ρ = 0.83, *p* < 0.001).

Peak heart rate responses during the 1MSTST and the 6MWT did not differ significantly (paired *t*-test, *p* = 0.160), while a strong positive correlation was observed between peak heart rate values achieved during the two tests (Pearson’s r = 0.75, *p* < 0.001; Figure 2a). Nadir SpO_2_ values did not differ significantly between the 1MSTST and the 6MWT (Wilcoxon signed-rank test, *p* = 0.919). A very strong positive correlation was observed between nadir SpO_2_ values obtained during the two tests (Spearman’s ρ = 0.83, *p* < 0.001; Figure 2b). Exercise-related oxygen desaturation (defined as a ≥4% drop from resting SpO_2_) occurred in 11 participants (20.4%) during the 1MSTST and 12 participants (22.2%) during the 6MWT. The proportion of participants meeting this criterion did not differ between tests (McNemar’s test, *p* = 1.00). Perceived dyspnoea increased significantly from rest following both the 1MSTST and the 6MWT (both *p* < 0.001). Modified Borg scale scores obtained after the two tests were strongly correlated (Spearman’s ρ = 0.76, *p* < 0.001). No statistically significant difference was observed between Modified Borg scale scores following the 1MSTST and the 6MWT (Wilcoxon signed-rank test, *p* = 0.058). The associated effect size was small (r = 0.26) (Table 4).

No adverse events occurred during testing. All participants completed both tests without interruptions.

## 4. Discussion

In this study of adults recovering from moderate to severe COVID-19, we found that performance in the 1 min sit-to-stand test (1MSTST) was moderately associated with distance walked during the 6 min walk test (6MWT), while cardiopulmonary responses—including peak heart rate and nadir SpO_2_—were strongly correlated between tests. Perceived exertion also showed substantial agreement. Together, these findings contribute to a growing body of evidence indicating that simplified field tests such as the 1MSTST capture overlapping aspects of functional capacity assessed by longer, standardized tests, while reflecting important physiological distinctions [17,18].

Although direct comparisons between the 1MSTST and the 6MWT in post-COVID-19 populations remain scarce, moderate to strong correlations between 1MSTST performance and 6MWT distance have been reported by available studies [19,20], supporting the concept that these tests assess related functional domains. Our findings extend existing literature by accounting for peripheral muscle strength and demographic and functional confounders.

The attenuation of the association between 1MSTST and 6MWT performance after adjustment for peripheral muscle strength highlights the distinct physiological determinants underlying each test. Sit-to-stand performance is strongly influenced by muscle strength and power, whereas walking-based tests depend more heavily on aerobic capacity, endurance, and cardiopulmonary integration [5]. Additional adjustment for age, sex, and post-COVID-19 functional status resulted in further attenuation and was no longer statistically significant, suggesting that shared patient characteristics rather than test equivalence accounted for much of the observed relationship.

Despite differing mechanical and temporal demands, the 1MSTST and the 6MWT elicited comparable cardiopulmonary responses, as reflected by similar mean peak heart rate and median nadir spO2 values and strong correlations in individual heart rate and oxygen saturation responses. Similar observations have been reported in patients with other chronic respiratory diseases, where shorter functional tests produced cardiovascular stress comparable to longer walking tests [9,10,11]. These findings support the physiological validity of the 1MSTST as a meaningful stimulus for cardiopulmonary assessment.

Most participants exhibited normal resting oxygen saturation and did not meet conventional criteria for exercise-induced hypoxemia. Nevertheless, neither the magnitude nor the frequency of clinically relevant oxygen desaturation (≥4% drop from rest) differed between the two tests. This pattern suggests that exertional desaturation primarily reflects individual physiological susceptibility—likely related to persistent pulmonary, vascular, or gas-exchange abnormalities after COVID-19—rather than a test-specific effect. The strong within-subject concordance in SpO_2_ responses supports the use of the 1MSTST for screening exertional desaturation when the 6MWT is not feasible.

Perceived exertion increased similarly following both tests, with strong correlations between post-test Borg scores. The small effect size observed for differences in Borg ratings suggests that both tests impose a comparable subjective burden, despite emphasizing different physiological components. This finding is consistent with previous reports comparing perceived exertion across functional field tests of differing duration and movement patterns [21].

Taken together, these findings align with existing recommendations emphasizing the value of simple, low-resource functional assessments in clinical practice, particularly in settings where space, time, or staffing constraints limit the feasibility of standardized walking tests. Beyond face-to-face evaluation, the 1MSTST has emerged as a particularly relevant tool for telerehabilitation in post-COVID-19 care. Its simplicity, minimal equipment requirements, absence of a clinically relevant learning effect, and short duration make it well suited for remote monitoring of functional status and therapeutic response [13].

Despite these contributions, the two tests should not be considered interchangeable. While the 6MWT remains the reference standard for detailed assessment of walking endurance and global functional capacity, the strong concordance observed in cardiopulmonary responses in the present study supports the role of the 1MSTST as a pragmatic complementary assessment, particularly within telerehabilitation pathways and in contexts where resource-intensive testing is not feasible.

Several limitations should be noted. First, convenience-based recruitment may have introduced selection bias toward individuals with better functional capacity and greater willingness to participate. Second, comorbidity data were not systematically collected in the original study database, which may limit adjustment for potential clinical confounders, nor did it include long-term follow-up to assess responsiveness or prognostic value. Third, our cohort reflects patients recovering in the early pandemic period, and findings may not generalize to later SARS-CoV-2 variants or to patients with milder disease phenotypes. Fourth, while handgrip dynamometry is a validated surrogate of overall muscle strength, the absence of direct lower-limb strength assessment may limit its ability to fully reflect the specific muscular demands of sit-to-stand performance. Fifth, the relatively preserved resting SpO_2_ in our sample may reduce sensitivity to detect exercise-induced desaturation in more severely desaturating populations. Finally, the single-center design and convenience sampling may limit external validity.

## 5. Conclusions

In adults recovering from moderate to severe COVID-19, the 1MSTST demonstrates moderate concordance with the 6MWT in functional performance and strong agreement in cardiopulmonary responses. These findings indicate that the two tests assess overlapping but distinct aspects of functional capacity. The 1MSTST may therefore serve as a practical, complementary assessment—particularly within telerehabilitation pathways and in primary care or resource-limited settings—when standard walking tests cannot be routinely implemented. Although the COVID-19 pandemic is now largely under control, the experience gained from functional exercise assessment in post-COVID-19 patients may provide a useful framework for evaluating recovery after other viral infections with respiratory tropism, a hypothesis that should be explored in future studies.

## Figures and Tables

**Table 1:** Exclusion criteria for this study.

Exclusion Criteria
Patients with a history of orthostatic hypotension
Patients with osteoarticular or neuromuscular diseases that affect standing or walking
Patients with unstable angina, myocardial infarction during the previous month, or arrhythmia
Patients with mental illness or cognitive impairment that prevents them from performing the examination safely and accurately
5. Who meet any of the following conditions
5-1. Pulse rate at rest ≤40/min or ≥120/min
5-2. Systolic blood pressure at rest ≤70 mmHg or ≥180 mmHg
5-3. Diastolic blood pressure at rest ≥100 mmHg
5-4. Complaining of palpitations, shortness of breath, chest pain at rest
5-5. Complaining of dizziness, cold sweat, or nausea in a sitting position at rest
6. Patients who are judged to be ineligible for 6MWT

**Table 2:** Baseline characteristics of study population.

Characteristic	Participants (n = 54)
Age (years), mean ± SD	57.9 ± 12.5
Male sex, n (%)	29 (54%)
Time from acute COVID-19 to assessment (months), mean ± SD	3.0 ± 2.4
Acute COVID-19 severity, n (%)	
• Moderate	32 (59%)
• Severe	22 (41%)
Post-COVID-19 Functional Status (PCFS), n (%)	
• Grade 0	9 (16.7%)
• Grade 1	16 (29.6%)
• Grade 2	23 (42.6%)
• Grade 3	6 (11.1%)
• Grade 4	0 (0%)
Handgrip strength (kg), mean ± SD	18.6 ± 9.2
1 min sit-to-stand test (repetitions), mean ± SD	20.5 ± 6.1
6 min walk test distance (m), mean ± SD	412 ± 90

Values are presented as mean ± standard deviation or number (percentage), as appropriate.

**Table 3:** Associations between functional performance measures before and after adjustment.

Analysis	Correlation Coefficient	*p*-Value
1MSTST repetitions vs. 6MWT distance	ρ = 0.47	<0.001
Hand grip dynamometry vs. 1MSTST repetitions	ρ = 0.49	<0.001
Hand grip dynamometry vs. 6MWT distance	ρ = 0.41	0.003
1MSTST vs. 6MWT (adjusted for peripheral muscle strength)	r = 0.35	0.012
1MSTST vs. 6MWT (adjusted for peripheral muscle strength, age, sex, PCFS)	r = 0.26	0.070

Spearman’s rank correlation coefficients (ρ) were used for unadjusted analyses. Partial correlation coefficients (r) were used for adjusted analyses.

**Table 4:** Cardiopulmonary and perceived exertion responses during the 1MSTST and the 6MWT.

Variable	1MSTST		6MWT		*p*-Value
Peak heart rate (bpm), mean ± SD	104.6	± 16.8	102.4	± 14.9	0.160
Nadir SpO_2_ (%), median (IQR)	96	(3)	96	(3)	0.919
≥4% SpO_2_ drop, n (%)	11	(20.4)	12	(22.2)	1.00
Perceived dyspnoea (Modified Borg scale, post-test), median (IQR)	5	(3)	4.5	(4)	0.058

Values are presented as mean ± standard deviation or median (interquartile range), as appropriate. Paired comparisons were performed using paired *t*-tests, Wilcoxon signed-rank tests, or exact McNemar tests, as appropriate.

## Data Availability

The data presented in this study are available on request from the corresponding author due to ethical reasons.

## Abbreviations

The following abbreviations are used in this manuscript:

6MWT	6-minute walk test
1MSTS	1-minute sit-to-stand test
SpO2	Peripheral oxygen saturation
HR	Heart rate
PCFS	Post-COVID Functional Status
COVID-19	Coronavirus disease 2019
SD	Standard deviation
IQR	Interquartile range
